# Endoscopic ultrasound-directed transgastric endoscopic retrograde cholangiopancreatography procedure for choledocholithiasis after sleeve gastrectomy and Roux-en-Y

**DOI:** 10.1055/a-2299-2127

**Published:** 2024-05-07

**Authors:** Reid D. Wasserman, Varun Kesar, Vivek Kesar, Paul Yeaton, Shehriyar Mehershahi

**Affiliations:** 1 246010 Department of Internal Medicine, Virginia Polytechnic Institute and State University Carilion School of Medicine, Roanoke, United States; 2 246010 Department of Gastroenterology, Virginia Polytechnic Institute and State University Carilion School of Medicine, Roanoke, United States

The endoscopic ultrasound (EUS)-directed transgastric endoscopic retrograde cholangiopancreatography (ERCP) procedure (EDGE) is employed when patients have altered anatomy from Roux-en-Y bypass surgery for treatment of biliary pathology. The procedure involves placing a lumen-apposing metal stent (LAMS) from the gastric pouch into the excluded stomach to create a fistula in which an ERCP scope can traverse to reach the ampulla 1 . However, patients who have previously undergone sleeve gastrectomy and Roux-en-Y bypass have a significantly smaller anatomical field to manipulate, which poses a technical challenge for the endoscopist. The case we report here shows that EDGE can be performed successfully using a gastro–gastric approach ( Video 1 ).

Endoscopic ultrasound-directed transgastric ERCP (EDGE) procedure for treatment of choledocholithiasis in a patient with a history of Roux-en-Y gastric bypass after sleeve gastrectomy.Video 1

The patient was a 65-year-old woman with a remote history of sleeve gastrectomy and subsequent Roux-en-Y gastric bypass surgery who originally presented to another hospital complaining of severe epigastric abdominal pain with associated nausea, vomiting, and fever. Initial lab investigations suggested ascending cholangitis. Computed tomography (CT) demonstrated biliary ductal dilatation secondary to a distal common bile duct stone. Follow-up magnetic resonance imaging revealed choledocholithiasis ( Fig. 1 ). Traditional ERCP could not be performed because of the patient’s history of Roux-en-Y gastric bypass surgery. As a result, the patient underwent CT-guided placement of an external biliary drainage catheter, which led to resolution of the cholangitis ( Fig. 2 ). She was discharged with plans for an outpatient EDGE procedure for definitive treatment. Two months later, the patient underwent an EDGE procedure with a 20 mm × 10 mm Axios stent (Boston Scientific, Marlborough, Massachusetts, USA) ( Fig. 3 , Fig. 4 , Fig. 5 ). Stent placement was followed by ERCP (4th generation Exalt D single-use duodenoscope; Boston Scientific) with successful biliary sphincterotomy and placement of a 10 mm × 4 mm fully covered metal stent (Boston Scientific) due to distal stenosis of the common bile duct. Both stages of the procedure were performed in a single session because the referral was to a facility several hours away. The patient had no complications and was discharged shortly after with plans to return in the near future for LAMS removal.

**Fig. 1:**
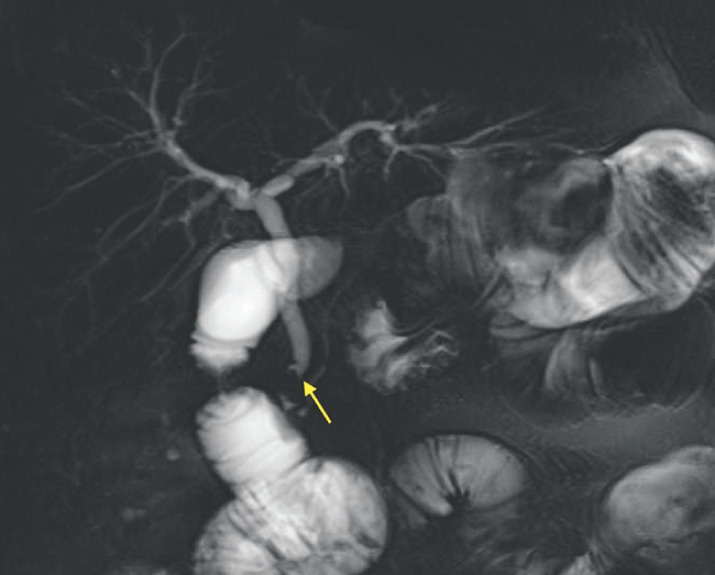
Magnetic resonance cholangiopancreatography in a 65-year-old woman with a remote history of sleeve gastrectomy and subsequent Roux-en-Y gastric bypass surgery, showing a filling defect consistent with a stone in the common bile duct.

**Fig. 2:**
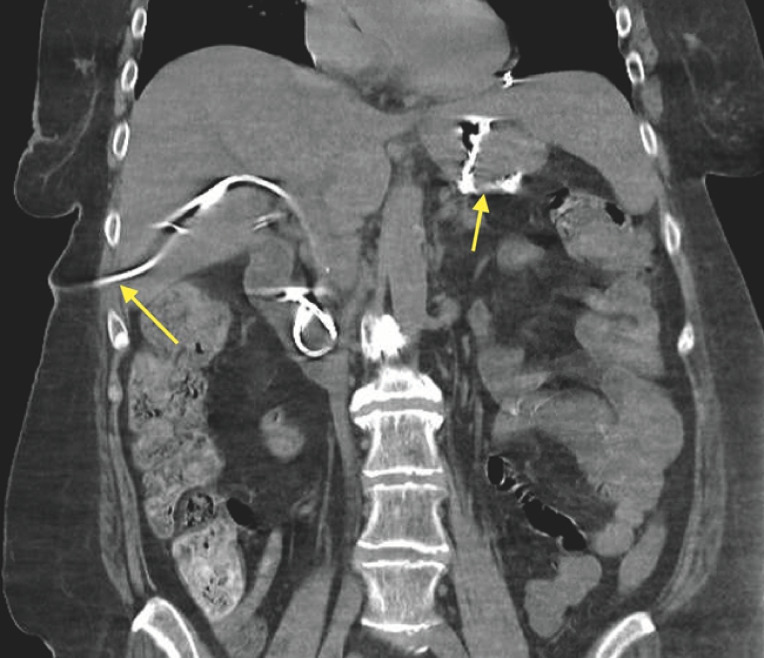
Computed tomography showing successful placement of a percutaneous biliary drain and gastric pouch (arrows).

**Fig. 3:**
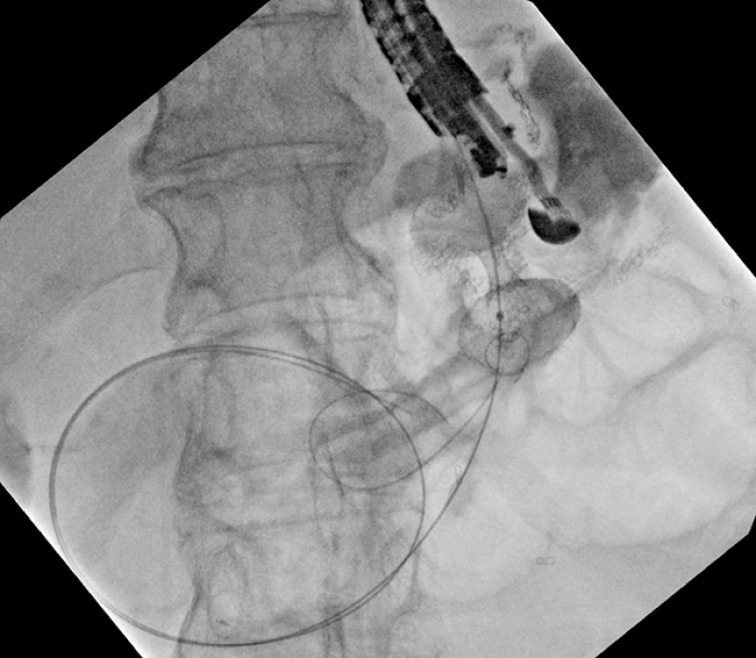
Fluoroscopic image of Axios lumen-apposing metal stent (LAMS) with guidewire passed through the gastro-gastric fistula.

**Fig. 4:**
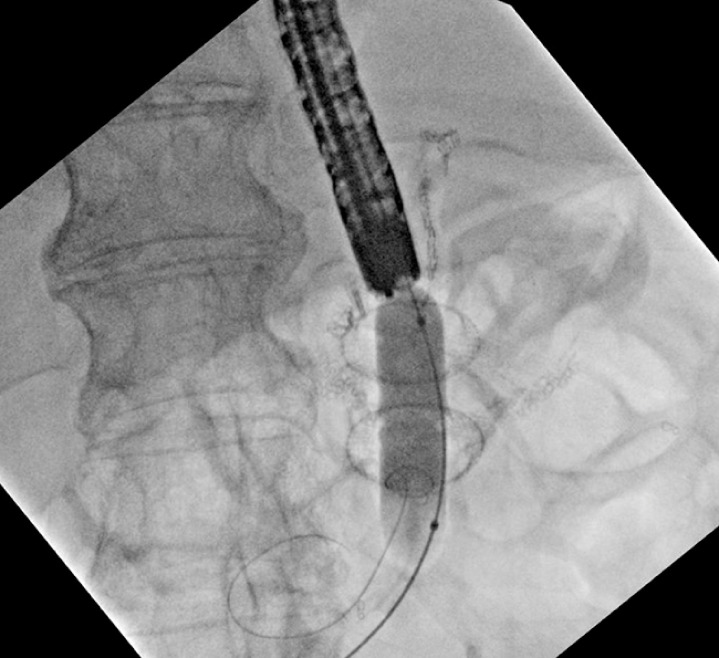
Fluoroscopic image of balloon dilation of the Axios LAMS creating a gastro-gastric fistula.

**Fig. 5:**
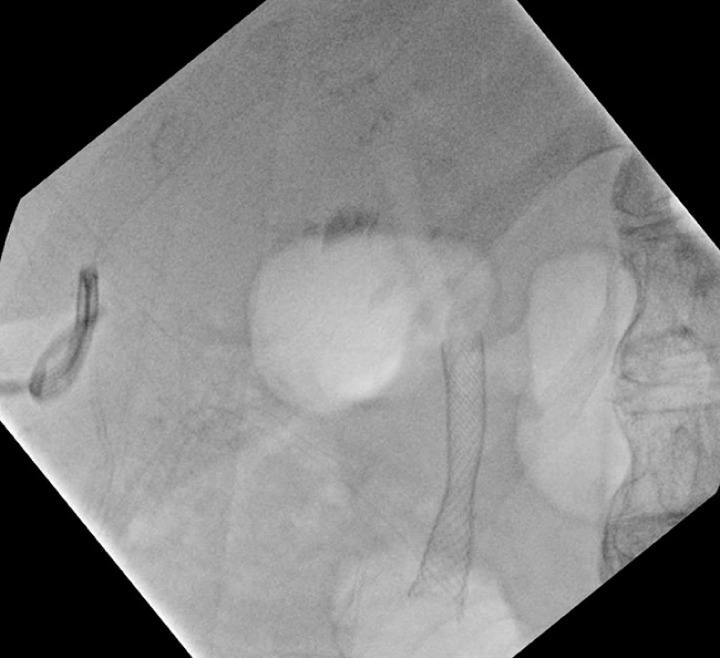
Fluoroscopic image of the Axios LAMS in final position creating a gastro-gastric fistula.

Endoscopy_UCTN_Code_TTT_1AS_2AD

## Notes

Endoscopy E-Videos https://eref.thieme.de/e-videos
*E-Videos* is an open access online section of the journal *Endoscopy* , reporting on interesting cases and new techniques in gastroenterological endoscopy. All papers include a high-quality video and are published with a Creative Commons CC-BY license. Endoscopy E-Videos qualify for HINARI discounts and waivers and eligibility is automatically checked during the submission process. We grant 100% waivers to articles whose corresponding authors are based in Group A countries and 50% waivers to those who are based in Group B countries as classified by Research4Life (see: https://www.research4life.org/access/eligibility/ ). This section has its own submission website at https://mc.manuscriptcentral.com/e-videos .
